# Editorial: Climate and Parasite Transmission at the Livestock-Wildlife Interface

**DOI:** 10.3389/fvets.2021.816303

**Published:** 2022-01-05

**Authors:** Nlingisisi D. Babayani, Hannah Rose Vineer, Josephine G. Walker, Rebecca K. Davidson

**Affiliations:** ^1^Okavango Research Institute, University of Botswana, Maun, Botswana; ^2^Department of Infection Biology and Microbiomes, University of Liverpool, Liverpool, United Kingdom; ^3^Department of Animal Health, University of Bristol, Bristol, United Kingdom; ^4^School of Social and Community Medicine, Norwegian Veterinary Institute, Tromsø, Norway

**Keywords:** climate, parasites, livestock-wildlife interface, cross-species transmission, one health, livelihoods, modeling, conservation

Livestock and wildlife parasites have profound effects on host populations and behavior. Parasitic diseases may restrict the ranges of host species, threaten the persistence of protected species, and alter trophic interactions, impeding conservation efforts, and production (1, 2). As pressure for shared resources grow, bringing wildlife, humans, and their livestock into closer and more frequent contact, there is increasing risk for spillover of parasites and parasitic diseases from wildlife to humans and livestock. Furthermore, most infectious diseases of humans, including parasitic diseases, are zoonotic, and wildlife maintain a considerable and unknown reservoir (3). This calls for a need to adopt a broader, multidimensional approach to veterinary parasitology research and parasitosis management. There is mounting evidence that wildlife-livestock interactions moderate parasite transmission, the epidemiology of parasitic disease, and the efficacy of parasite control programmes (4), and this is more pertinent at the wildlife-livestock interface (5). Given that the development and survival of many parasites are intimately linked with the environment, climate change may alter environmental suitability for these species, affecting their abundance and seasonal population dynamics. Climate change may also alter the behavior and distributions of host species, impacting on host–parasite interactions and affecting transmission dynamics (6, 7).

The current true global scale and impacts of parasite and parasitic diseases transmission at the wildlife–livestock interface is not known and in the few localities where it has been adequately explored, generalizability to other localities across the globe is difficult because of variability in climatic conditions, anthropogenic activities, and species concerned. To date, significant resources have been devoted to developing knowledge, tools, and parasite control strategies to optimize livestock productivity for the benefit of mankind and to improve animal health and welfare. Many of these studies treat livestock farming systems as “monocultures,” largely with single species parasitic infections, yet livestock exist within the wider ecological community, which includes sympatric wildlife and coinfection. By taking a broader and multidimensional approach to veterinary parasitology, we may identify new opportunities for interventions to reduce parasitism, or conversely, the coexistence of livestock and wildlife for mutual benefit, with potential to enhance access to ecosystem services and improve livelihoods. Understanding how wildlife and livestock interact, host–parasite networks, and how these systems exist within the wider environment could deliver benefits for sustainable control of endemic parasites, optimal livestock production, invasive parasite control, conservation, and human well-being. This Research Topic is aimed at accumulating research findings that explore parasite transmission at the livestock–wildlife interface, worldwide, with a particular emphasis on transmission dynamics in mixed host–species communities, and how climate alters these dynamics. In addition to shedding more light on parasite transmission at varied global livestock–wildlife interfaces, this topic is expected to highlight the “One Health” concept as applied to wildlife and livestock, and promote a unifying, ecological approach to veterinary parasitology.

In this special issue, there are five papers documenting various parasite species and their transmission at environmentally different wildlife–livestock interfaces and land use backgrounds. One paper documented the first report of a zoonotic parasite species, *Macracanthorhynchus hirudinaceus*, in feral pigs in a natural park of Sicily (Southern Italy), where sympatric farming of domestic pigs is also practiced (Migliore et al.). The paper discusses the potential implication of spillover from the wildlife interface to domestic pigs, with increasing extensive farming practices that have limited biosecurity measures, and human health. Two articles assessed cross-transmission of nematodes between wildlife and co-grazing livestock in mixed-use landscapes, in the context of prevailing climatic conditions for parasite existence (Khanyari et al.; Rose Vineer et al.). Khanyari et al. explored gastro-intestinal nematodes (GINs) infections, in general, between livestock and Bharal in the North India Trans-Himalayas from a socio-ecological perspective by integrating mechanistic parasite transmission modeling with field surveys and local knowledge. The models evaluated the likely effectiveness of potential interventions needed to support herders' livelihoods and conserve wild ungulates and thus help herders develop successful intervention strategies. Rose Vineer et al. assessed the impact of recent climate change on the reindeer brain worm (*Elaphostrongylus rangiferi*), by modeling thermal suitability for its transmission in Fennoscandia (Norway, Sweden, and Finland) to estimate potential risk to the health of semi-domesticated and wild reindeer, as well as co-grazing small ruminants, and therefore the threat to livelihoods of indigenous herders. This preliminary model helps reindeer herders and small ruminant farmers identify periods with potential high infection risk to evaluate if mitigation strategies are needed. The last two articles looked at potential for vector-borne parasite (tick-borne pathogens and trypanosome species) transmission dynamics between wildlife and domestic stock in adjacent landscapes of distinct use, one for livestock rearing and another for wildlife conservation (Babayani and Makati; Kalayou et al.). Babayani and Makati identified important drivers of tick distribution in cattle in the lower Okavango Delta, Botswana. Such knowledge is needed to enable forecasting of the risk of tick-borne diseases in the region. Kalayou et al. updated the epidemiological data regarding trypanosomiasis at the wildlife-livestock interface in Lambwe, Kenya, and demonstrated that human African trypanosomiasis has likely been eliminated from this region, but that bovine trypanosomiasis remains a significant problem, especially for herders in close proximity (<2 km) to a national park.

In summary, presence and expansion of wildlife in areas close to human settlements, coupled with farming of domestic stock at such an interface allows for uncontrolled contact, hence potential veterinary and public health threat for cross-species transmission (8). This may lead to serious socio-economic consequences through diminished productivity in domestic stock because of disease outbreaks and risk to human health from zoonotic diseases (5, 9). Since all interfaces cannot be considered important from a parasite transmission perspective, there is a need for surveillance and research approaches that target specific wildlife–livestock interfaces for better understanding to inform adoption of resilience strategies in the face of climate change.

## Author Contributions

NB drafted the initial editorial manuscript. HR commented on the central theme of the editorial. RD commented on the research topic findings. JW submitted comments on the introductory remarks and conclusion. All authors contributed to the article and approved the submitted version.

## Conflict of Interest

The authors declare that the research was conducted in the absence of any commercial or financial relationships that could be construed as a potential conflict of interest.

## Publisher's Note

All claims expressed in this article are solely those of the authors and do not necessarily represent those of their affiliated organizations, or those of the publisher, the editors and the reviewers. Any product that may be evaluated in this article, or claim that may be made by its manufacturer, is not guaranteed or endorsed by the publisher.
